# Cheyne-Stokes respiration in patients hospitalised for heart failure

**DOI:** 10.1186/1465-9921-5-14

**Published:** 2004-09-20

**Authors:** Lena Mared, Charles Cline, Leif Erhardt, Søren Berg, Bengt Midgren

**Affiliations:** 1 Dept of Respiratory Medicine, University Hospital, Lund, Sweden; 2 Dept of Cardiology, University Hospital, Malmö, Sweden; 3 Dept of ENT diseases, University Hospital, Lund, Sweden; 4 Lund Sleep Study Group, University Hospital, Lund, Sweden

## Abstract

**Background:**

Previous studies showing a strong relationship between Cheyne-Stokes respiration and the severity of left ventricular systolic dysfunction have usually been done in selected patient populations with lower age and a higher proportion of males than the "typical" in-hospital patient with heart failure. The purpose of the present study was test the strength of this relationship in unselected patients admitted to hospital due to decompensated chronic heart failure.

**Methods:**

We evaluated 191 patients (32% women), mean age 73 years, ready for discharge from the heart failure unit in the University Hospital of Malmö, Sweden. The patients underwent echocardiography for determination of left ventricular ejection fraction and left ventricular inner diastolic diameter. A respiratory investigation during sleep was performed the last night before discharge.

**Results:**

We found that 66% of the patients had Cheyne-Stokes respiration more than 10% of the total recording time. Only 7 (3.6%) of the patients had predominantly obstructive apnoeas. There was a significant but very weak relationship between left ventricular ejection fraction and left ventricular inner diastolic diameter on one hand and Cheyne-Stokes respiration on the other. Age was a stronger determinant of Cheyne-Stokes respiration than any of the cardiac or other clinical variables.

**Conclusion:**

Although presence of Cheyne-Stokes respiration indicates left ventricular dysfunction, its severity seems only weakly related to the severity of heart failure. Age was found to be a stronger determinant, which may reflect the underlying age-dependency found also in healthy subjects. Due to age restrictions or other selection criteria, the importance of age may have been underestimated in many previous studies on factors associated with Cheyne-Stokes respiration.

## Background

Cheyne-Stokes respiration (CSR) during sleep, is common in patients with heart failure [1,2]. Cheyne-Stokes respiration during sleep has been claimed to be an independent risk factor for death [3,4], speculatively through increased neurohumoral stress on the heart [5]. Results from other studies have, however, been contradictory [6]. It has also been claimed that sleep disturbance from Cheyne-Stokes respiration may cause daytime sleepiness [7].

Most previous studies have included selected patients with impaired left ventricular systolic function. A large proportion of heart failure patients are elderly and have relatively preserved left ventricular systolic function. However, elderly heart failure patients have often been excluded from sleep studies. Such studies are not representative of the everyday clinical spectrum of heart failure patients, including the increasing number of elderly patients usually seen in general medical wards. The proportion of women, with predominantly diastolic heart failure, may also be greater in this group.

The aims of the present study were to test the strength of the relationship between left ventricular dysfunction and nocturnal Cheyne-Stokes respiration in unselected patients admitted to hospital due to decompensated chronic heart failure and to determine the correlation between Cheyne-Stokes respiration and other clinical variables to provide source material for subsequent analyses of quality of life and survival.

## Methods

### Patients

All patients admitted to the Heart Failure Unit at the Department of Cardiology, Malmö University Hospital from January 1996 to november 1999 primarily due to decompensated chronic heart failure were eligible for inclusion. Malmö University Hospital serves as the main hospital for the whole population of Malmö (population 250,000) and is the only hospital in the city. Patients were excluded only if unable to comply to the study protocol due to some other condition, to complete the study questionnaire or to provide informed consent. Heart failure was diagnosed according to European Society of Cardiology guidelines for the diagnosis of heart failure [8]. All patients had been stabilised following treatment for heart failure and were studied on the day prior to planned discharge. For practical reasons not all patients discharged could be included into the study. Patients were included if they were to be discharged on a weekday and only when a nurse trained in the use of the registration apparatus was available. Furthermore, we could not register more than one patient per night. Unfortunately, the precise number of patients discharged alive from this unit during this particular period was not possible to obtain, but a crude estimate is that we have investigated between 25 and 50% of the available patients.

All patients provided informed consent to participate in the study, which was performed in accordance with the principles of the Declaration of Helsinki and approved by the Medical Ethics Committee at Lund University.

### Clinical evaluation

Ischaemic heart disease was diagnosed based on findings from previous coronary angiography, documented myocardial infarction or typical signs of ischaemia at exercise testing. Hypertension (according to the local guidelines at the time of the study) was diagnosed if blood pressure was >150/95 mmHg or the patient was receiving drug therapy for hypertension. Diabetes was diagnosed if fasting blood glucose levels were >7 mmol/l or the patient was treated with oral anti-glycaemic medication or insulin. The diagnosis of other concomitant diseases was based on patient history and/or patient records.

### Echocardiography

Echocardiographic examinations were performed using a Hewlett-Packard Sonos 2000 (Andover, Mass, USA). Parasternal and apical views were obtained with the patient in a left lateral recumbent position. Measurements were acquired during silent respiration or end-expiratory apnoea. Left ventricular systolic function was assessed by determination of the mean left atrioventricular plane displacement (AVPD), global qualitative assessment and/or single plane ellipse (modified Simpson's rule) [9-11].

### Respiration during sleep

We recorded oronasal airflow by thermocouples, electrocardiogram, chest wall movement by electrical impedance, and finger pulse oximetry using the EdenTrace II Plus Multirecording System (EdenTech Corp, Eden Prairie, MN, USA) [12,13]. The recordings started when the patients went to bed and were discontinued the following morning when the patients woke up. No attempt was made to define the amount of sleep. The recordings were printed out and scored manually. Hypnotics were allowed, and were taken by 48% of the patients at the time of the study. Patients were considered to be habitual snorers if they answered "often" or "always" to the question "Do you snore loudly and disturbingly?"

### Scoring and analysis of breathing patterns

The records were scored manually for Cheyne-Stokes respiration (gradual waxing and waning of respiration followed by a central apnoea or hypopnoea) [14,15] and for obstructive sleep apnoea. Patients with purely or predominantly obstructive sleep apnoea (n = 7) were excluded from the analyses of CSR. Patients with occasional obstructive apnoeas occuring during extended periods of CSR (n = 28) were included in the analyses of CSR. The total time spent in Cheyne-Stokes respiration was divided by the total recording time to compute the percentage of time in bed spent in Cheyne-Stokes respiration (CSR%).

Since CSR% was not normally distributed, we used Spearmans rank correlation test to relate CSR% to the continuous variables age, BMI, LVEF and LVIDD. For analysis of CSR% with respect to the categorical variables gender, cerebrovascular disease, ischaemic heart disease, NYHA class, atrial fibrillation and habitual snoring, we used the Mann-Whitney U-test.

There are no data in the literature to allow categorisation of patients as normal and abnormal according to any specific level of CSR%. In table 2, the data concerning CSR% are, however, categorised; this is for demonstrational purpose only.

**Table 2:** Cheyne-Stokes respiration related to physiologic variables

	All CSR incl mixed apnoeas			
		
	CSR%	CSR%	CSR%	Significance
	<10	10–50	>50	
	n = 58	n = 66	n = 60	
Age (years)	68.8 ± 11.7	72.1 ± 8.5	75,9 ± 8.6	p < 0.01
				R=0.24
Body Mass Index (kg/m^2^)	26.5 ± 5.5	26.1 ± 4.3	24.6 ± 4.1	NS
LVEF (%)	36.1 ± 12.1	38.8 ± 12.6	32.4 ± 11.5	P < 0.05
				R=-0.17
LVIDD (mm)	55.1 ± 10.0	57.1 ± 9.4	58.0 ± 8.2	P < 0.01
				R = 0.20
Ischaemic heart disease (percentage of patients)	51	59	72	p < 0.05
NYHA class 3–4 (percentage of patients)	60	39	55	NS
Atrial fibrillation (percentage of patients)	29	33	40	NS
Cerebrovascular disease (percentage of patients)	16	11	27	NS
Habitual snorers (percentage of patients)	12	12	12	NS
Males (percentage of patients)	57	77	70	NS

Values are given as mean ± SD. Patients are divided according to the severity of Cheyne-Stokes respiration (quantified as percentage of total recording time); the limits are arbitrarily chosen. Significance testing is made with Spearman's rank correlation test for continuous variables and with Mann-Whitney U-test for categorical data.

## Results

### Clinical data

Two hundred and three patients were included, however final analysis only included 191 patients (32% women). Three patients were excluded because of total recording time less than two hours. Eight patients were excluded due to the poor quality of their recordings and one patient was excluded because of an abnormal irregular breathing pattern that could not be categorized as Cheyne-Stokes or obstructive sleep apnoea.

The aetiology of the heart failure was ischaemic heart disease in 60%, dilated cardiomyopathy in 3%, hypertension in 14%, valvular disease in 8% and other or unknown reason in 15%. Half of the patients had had heart failure diagnosis for more than a year. All but eight patients were prescribed diuretics, 43% digitalis, 70% ACE inhibitors and 28% beta-blockers. Almost half (48%) of the patients used hypnotics (usually bensodiazepines).

The clinical characteristics of the study patients are presented in Table 1. Five patients were in NYHA class 1, 83 in NYHA 2, 89 in NYHA 3 and five in NYHA 4. Only 10 patients were free from concomitant disease. Twenty five percent had diabetes, 13% chronic obstructive pulmonary disease and 18% cerebrovascular disease (reversible or permanent cerebral ischaemia or haemorrhage) at any time prior to the investigation. Five percent had cancer diagnosed and treated within the last year.

**Table 1:** Clinical characteristics of the study patients

	All patients	Females (n = 61)	Males (n = 130)	Significance
Age (years)	72. 6 ± 10.0	75.1 ± 8.9	71.4 ± 10.3	P < 0.05
BMI (kg/m^2^)	25.7 ± 4.6	25.4 ± 5.4	25.9 ± 4.3	NS
LVIDD (mm)	56.5 ± 9.4	51.2 ± 9.5	59.0 ± 8.3	P < 0.001
LVEF (%)	36.2 ± 12.1	39.0 ± 13.0	34.9 ± 11.6	P < 0.05
LVEF ≥45% (percentage of patients)	26	36	22	P < 0.05
Ischaemic heart disease (percentage of patients)	60	57	62	NS
NYHA 3–4 (percentage of patients)	52	57	49	NS
Atrial fibrillation (percentage of patients)	35	33	35	NS
Cerebrovascular disease (percentage of patients)	18	15	19	NS
Habitual snorers (percentage of patients)	12	7	15	P < 0.05

Values for continuous data (age, BMI, LVIDD and LVEF) are given as mean ± SD. Significance testing is made with T-test for continuous data and chi-square for the other data.

Overall 26% had heart failure due to left ventricular diastolic dysfunction (ejection fraction >45%). Female patients were older (Table 1) and more likely to have heart failure with preserved left ventricular systolic function; 36% had ejection fraction ≥45% as compared to 22% of the males (p < 0.05, chi square). There was no significant gender difference in the prevalence of ischaemic heart disease as the cause of heart failure.

### Respiration during sleep

**Figure 1:**
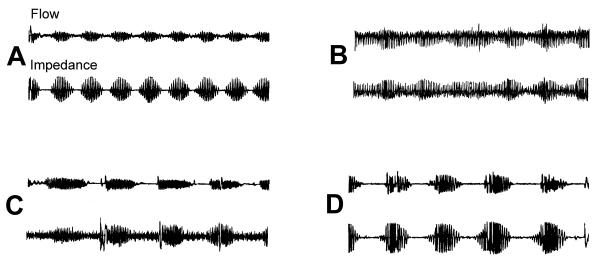
**Examples of nocturnal respiratory recordings. **The upper part of each panel is the flow signal from the thermocouples and the lower is the impedance signal from the ECG electrodes. The duration of each example is 6 minutes. Panel A depicts an unequivocal period of Cheyne-Stokes respiration. Panel B was interpreted as normal by the software of the recording device, but was interpreted by us as Cheyne-Stokes respiration. Panel C is an example of obstructive sleep apnoeas and panel D is a period of Cheyne-Stokes respiration with a small obstructive component, classified by us as Cheyne-Stokes respiration rather than obstructive sleep apnoeas.

The average recording time was 424 (SD 75, median 444, interquartile 400–472) minutes and average CSR% 35 (SD 30, median 28, interquartile 6–59). Predominantly obstructive apnoeas were found in seven patients (3.7%) and CSR (arbitrarily defined as CSR% >10%) in 126 patients (66%). Sixty (31%) of the patients had CSR more than 50% of the recording time (Table 2). In figure 1, four examples of different breathing patterns are demonstrated.

**Figure 2:**
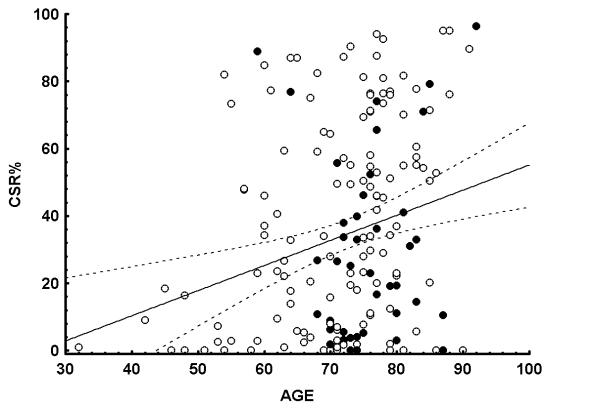
**Cheyne-Stokes respiration as a function of age. **Cheyne-Stokes respiration (% of total recording time) as a function of age, linear regression line and confidence bands are drawn. Filled circles denote patients with LVEF 45 and above, i.e. patients with diastolic heart failure.

Univariate rank correlation analysis showed that CSR% was most strongly correlated to age (R = 0.24, p < 0.01, figure 2), but also to left ventricular ejection fraction (LVEF) and to left ventricular diastolic diameter (table 2). Stepwise multiple regression analysis with gender, age, BMI, LVEF and LVIDD as independent variabels confirmed the relative importance of age vs that of LVEF and LVIDD (r^2 ^0.06 vs 0.03 and 0.03). The severity of CSR was also greater in patients with ischaemic heart disease. There were no relationships between medication (e.g. betablockers or benzodiazepines) and CSR%.

Seven patients (3.6%) were found to have predominantly obstructive sleep apnoea (average AHI 16.0, SD 7.0, median 16.2, interquartile range 8.7 – 21.8). Excessive snoring, obesity or male gender were not overrepresented in this group.

## Discussion

We have shown that CSR is common in elderly patients hospitalised due to decompensated chronic heart failure and that age was a stronger determinant of CSR than any of the cardiac or other clinical variables

### Selection of patients and timing of the study

Malmö University Hospital serves a population of 250,000 inhabitants. In general all patients primarily admitted to hospital due to decompensated chronic heart failure are treated at the Heart Failure Unit until discharge from hospital. Due to this and to the liberal inclusion criteria we believe that the study patients are representative of the general population of patients with heart failure. Many other studies suffer from the disadvantages of a selection bias by excluding older patients, including only men or including only patients specifically referred for sleep studies [16] or for evaluation for heart transplantation [17].

One major difference between our study design and that of the majority of other studies is that we included patients immediately after an episode of decompensation, whereas most other authors have studied patients 1–3 months after discharge. We do not, however, know to what extent this approach affects nocturnal breathing patterns. The results reported by Tremel et al [2] suggest that sleep respiratory disturbances are stable during the second month after an episode of worsening heart failure, whereas there are no data, as far as we know, examining patients prior to that phase. Nonetheless, many observations of apnoeas (by nurses and relatives, posing questions to the clinician) are made during the hospitalisation. Our results are therefore relevant with respect to the factors associated with apnoeas in this situation.

### Respiratory disturbances during sleep

At the time of the study, the most convenient and easy manageable system available at our department was the EdenTrace system. The semiquantitative nature of chest wall impedance measurement may have reduced the sensitivity of our measurements. However, by combining impedance measurement of respiratory movements, recording of oronasal airflow with thermocouples and finger pulse oxymetry with a careful visual analysis af the traces by an experienced physician (SB), we postulate that the distinction between central and obstructive events is sufficiently accurate. Furthermore that the bias introduced, would tend to underestimate, rather than overestimate the prevalence of sleep disordered breathing in the studied population.

Whereas there are widely accepted standards for analysis of sleep apnoea the definition of CSR is less precise, and there is no accepted standard method for its quantification. We quantified the severity of CSR as percentage of total recording time. This approach is suggested by Ancoli-Israel et al [14] and is accepted also by the American Academy of Sleep Medicine Task Force [15] since it is simple and well suited for routine clinical use, irrespective of the technical methods available for respiratory recordings.

The overall prevalence of CSR in this material was 66%, using an arbitrary limit of CSR% >10%. This figure is in the same magnitude as the 50–60% previously reported in stable heart failure outpatients [18,19] and close to that found by Ancoli-Israel (70%) in a small group of elderly, hospitalised patients [14]. We suggest that the main cause of the prevalence differences between studies is the age of the population studied, rather than the timing with regard to worsening heart failure [2].

We found 28 patients with mixed apnoeas (see figure 1 panel D for example). We believe that these patients should be considered to have a variant of CSR rather than to have an obstructive sleep apnoea syndrome (OSAS). There was no excess of snoring, obesity or male gender in this group, factors that are otherwise considered to be associated with OSAS. Furthermore, exclusion of these patients from the analyses did not change our results. It has been suggested that obstructive sleep apnoeas and CSR in heart failure patients both are part of a spectrum of periodic breathing [20,21], our data are compatible with this hypothesis.

### Age

The most consistent result of our study is that age was more strongly related to Cheyne Stokes respiration than any other variable recorded. This corroborates data from other large studies without an upper age limit [14,16,22]. Many other studies that failed to demonstrate a similar relationship are constrained to patients below an arbitrary age limit or to patients referred for cardiac transplantation [17], which strongly affects validity for the general in-hospital patients. The strength of the association with age remained also when we excluded the youngest outliers (see figure 2) from the statistical analysis. We therefore consider our finding of age-dependency to be valid for unselected in-hospital patients with heart failure. It should be emphasized, however, that the predictive value of age was very weak, only explaining 6% of the total variability.

Increasing prevalence of central apnoeas with age in normal subjects has been demonstrated by e.g. Bixler and coworkers [23], although the prevalence is much lower than that found in our patients with heart failure. Bixler et al suggest that a conservative approach should be applied when interpreting sleep studies in elderly. Our data suggest that this may also be a valid strategy for patients with heart failure. Although the presence of CSR seems to be associated with the presence of heart failure, its severity gives little information about the severity of the heart disease.

### Gender

We found no gender effect (table 2), in contrast to the findings of *e.g*. Sin and coworkers [16]. One important reason may be that their 450 patients (only 15% women vs 32% in our study) were not a random sample of heart failure patients, but represent a much younger population (mean age 60 years vs 72 in our study) specifically referred for a sleep study.

### Indices of heart failure

Heart dilatation (in our study measured as LVIDD) has been claimed to be an important factor for the development of CSR [24]. Impaired systolic function, as demonstrated by a low LVEF, is another factor that is usually considered to be associated with CSR. In our data, we found that these factors explained only 3% each of the variability of CSR. This contrasts to the results of many other studies, but is well in accordance with the findings of Sin and coworkers in their large study [16]. The idea of CSR mainly being a function of low cardiac output (as estimated by e.g. LVEF) may therefore be an oversimplification.

Atrial fibrillation is a third factor that has been claimed to be associated with CSR [16,18,22], but this could not be confirmed in our study. One reason for the discrepancy may be that the patients in the studies of Sin [16] and Javaheri [18] were considerably younger than our patients, with a lower prevalence of atrial fibrillation. Thus their findings may have been confounded by an age effect. However the study of Blackshear [22] with a non-selected sample of elderly heart failure patients with a high prevalence of atrial fibrillation demonstrates a strong relationship between atrial fibrillation and CSR. The reason for the discrepancy between their results and our present study is not obvious.

### Concomitant diseases

Patients with a history of stroke are usually excluded from studies on CSR and heart failure. This may be a limitation of the external validity of such studies, since many patients with heart failure (18% in our material) also have a history of minor or major cerebrovascular disease. We found however, quite unexpectedly, that this was not associated with a higher occurrence of CSR, thereby corroborating the findings of Blackshear et al [22]. Neither was any other concomitant disease associated with CSR. There was no association between intake of hypnotics and CSR.

## Conclusions

Although presence of Cheyne-Stokes respiration indicates presence of left ventricular dysfunction, its severity seems only weakly related to severity of heart failure. Age was a stronger determinant, which may reflect the underlying age-dependency found also in healthy subjects. Follow-up of the current patient cohort will be performed, but from the present data, we cannot conclude if Cheyne-Stokes respiration is of clinical importance or not.

## Authors' contributions

LM coordinated and performed the study which was designed by CC and BM. LE and CC were responsible for the cardiac investigations and SB and BM for the interpretation of the nocturnal respiratory recordings.
